# Physicochemical indicators and recurrence of urolithiasis in patients with hyperuricemia: a predictive model

**DOI:** 10.1186/s12894-026-02273-4

**Published:** 2026-08-12

**Authors:** Ziyi Li, Zhaoheng Jin, He Xiao, Zhi Gang Ji, Yi Qiao, Lin Ma

**Affiliations:** 1 Chinese Academy of Medical Sciences & Peking Union Medical College (CAMS & PUMC), Peking Union Medical College Hospital, Beijing, China; 2 Department of Urology Surgery, Peking Union Medical College Hospital, Chinese Academy of Medical Sciences and Peking Union Medical College, Beijing, China

**Keywords:** Urolithiasis, Recurrence, Hyperuricemia, Urinary Citrate, Prediction Model

## Abstract

**Objective:**

To identify metabolic predictors of urolithiasis recurrence and investigate whether the predictive value of urinary citrate differs across metabolic subgroups, with the aim of developing a subgroup-specific prediction model.

**Methods:**

We retrospectively analyzed patients who experienced a first episode of urolithiasis between 2013 and 2015 and subsequently underwent metabolic evaluation at the Department of Urology of our institution. Baseline assessment included demographic characteristics, blood biochemical parameters, and 24-h urinary metabolic indices. Follow-up data on stone recurrence were collected through standardized telephone interviews conducted between 2023 and 2025. Multivariable logistic regression and subgroup analyses were performed to identify predictors of recurrence. A prediction model for patients with hyperuricemia was developed using stepwise logistic regression and internally validated by tenfold cross-validation.

**Results:**

A total of 184 patients were included. Multivariable logistic regression identified 24-h urinary citrate as the only metabolic parameter independently associated with stone recurrence (*P* = 0.04). Correlation analysis demonstrated significant associations between urinary citrate and serum uric acid, urinary calcium, urinary oxalate, and age. Subgroup analysis showed that the inverse association between urinary citrate and recurrence remained significant only among patients with hyperuricemia (*P* = 0.03), whereas similar trends in other metabolic subgroups did not reach statistical significance. The interaction between serum uric acid and urinary citrate was not statistically significant (*P* = 0.40). A preliminary prediction model incorporating urinary citrate, age, BMI, triglycerides, and parathyroid hormone demonstrated moderate discriminative performance, with a mean cross-validated AUC of 0.747.

**Conclusion:**

Urinary citrate was independently associated with urolithiasis recurrence, with this association being particularly evident among patients with hyperuricemia. These findings suggest that the predictive value of urinary citrate may vary across different metabolic backgrounds and support further investigation of metabolically stratified approaches to recurrence risk assessment. External validation in larger prospective cohorts is warranted.

**Supplementary Information:**

The online version contains supplementary material available at https://doi.org/10.1186/s12894-026-02273-4.

## Introduction

Urolithiasis is a prevalent global health issue that imposes a substantial economic burden on healthcare systems worldwide [1, 2]. The formation of urinary tract stones occurs when the balance between stone-promoting substances and crystallization inhibitors in the urine is disrupted. Key promoters of stone formation include calcium, oxalate, phosphate, bacterial byproducts, cystine, low urine volume, uric acid, and acidic urine pH, whereas inhibitors such as citrate and magnesium help reduce the risk of crystal formation. Besides its widespread prevalence, urolithiasis also demonstrates a high recurrence rate, with up to 50% of patients experiencing recurrence within five years of their first stone episode [3, 4].

Previous studies have shown that several urinary components are associated with stone formation. For instance, urinary excretion of calcium, oxalate, and phosphate directly affects the supersaturation of calcium oxalate and phosphate, while other elements, including citrate, uric acid, magnesium, potassium, sodium, and urine pH, may exert indirect effects [5]. Since the 1980 s, increasing attention has been paid to pharmacological interventions for the prevention of stone recurrence. Citrate, a well-established inhibitor of stone formation, binds free urinary calcium ions, thereby reducing calcium oxalate crystal formation [6], potassium citrate therefore become an established pharmacological therapy for recurrent stone formers.

Although metabolic evaluation has identified numerous physicochemical parameters associated with stone formation, previous longitudinal studies have demonstrated that most routine metabolic variables have limited ability to independently predict stone recurrence, whereas urinary citrate has consistently been identified as one of the few metabolic parameters associated with recurrence risk [7, 8]. Hyperuricemia is a well-recognized metabolic abnormality associated with urinary acidification and an increased risk of stone recurrence, suggesting that the predictive value of urinary citrate may differ in this high-risk population. However, whether the predictive value of urinary citrate differs among patients with different metabolic phenotypes remains largely unknown. Given the heterogeneity of metabolic abnormalities among stone formers, identifying subgroup-specific predictors may improve individualized risk stratification. Therefore, this study aimed to evaluate the association between physicochemical parameters and long-term stone recurrence, with particular emphasis on whether urinary citrate exhibits differential predictive value across metabolic phenotypes. Based on these findings, we further developed a preliminary subgroup-specific prediction model for patients with hyperuricemia.

## Materials and methods

### Study population

This single-center retrospective observational study initially screened a total of 460 consecutive patients who presented to Peking Union Medical College Hospital with a first episode of urolithiasis between January 2013 and December 2015. At the initial presentation, all patients were considered first-time stone formers. Follow-up was conducted annually between 2023 and 2025 through structured telephone interviews using a standardized questionnaire. During follow-up, information regarding stone recurrence and the cumulative number of stone episodes after the initial presentation was collected using the standardized questionnaire. Patients were excluded if they met any of the following criteria after their initial visit: long-term use of anti-lithogenic medications (e.g., citrate-based agents, uric acid-lowering therapies, sodium bicarbonate), chronic use of medications that could influence metabolic assessment (e.g., lipid-lowering drugs, antihypertensive agents, calcium supplements, antidiabetic medications, vitamin D), significant changes in water intake (± 1000 mL), significant changes in body weight (± 10 kg), or had undergone thyroid, urinary tract, or gastrointestinal surgery. Information on medication use, significant changes in water intake and body weight, and interval surgical history was collected during the structured telephone interviews. After application of the eligibility criteria, 184 patients were included in the final analysis.

### Independent variables

All independent variables were obtained from baseline metabolic evaluations performed at the patients' initial visit. The evaluated variables included age, body weight, height, glycated hemoglobin (HbA1c), triglycerides (TG), high-density lipoprotein cholesterol (HDL-C), low-density lipoprotein cholesterol (LDL-C), serum uric acid, parathyroid hormone (PTH), urinary α1-microglobulin, 24-h urinary oxalate, citrate, calcium, and HOMA-IR. Blood biochemical parameters, including HbA1c, TG, HDL-C, LDL-C, serum uric acid, and PTH, were measured in the Clinical Laboratory of Peking Union Medical College Hospital using routine clinical laboratory procedures. Twenty-four-hour urine samples were collected according to routine clinical instructions, and urinary biochemical parameters, including urinary oxalate, citrate, calcium, and α1-microglobulin, were analyzed using standardized laboratory protocols.

### Outcome variable

The primary outcome was stone recurrence during follow-up, which was assessed using the following standardized question:

*“Since your last visit, have you experienced spontaneous stone passage or received a diagnosis of urinary tract stones by any imaging modality?”*

Previous studies have demonstrated the reliability of self-reported urolithiasis recurrence [8]. Participants who answered "yes" were considered to have experienced stone recurrence. Patients with ≥ 2 stone episodes over the 10-year period were categorized as having experienced stone recurrence. This binary outcome variable (Stone_Group) was used as the dependent variable in subsequent analyses.

### Statistical analysis

#### Logistic regression and correlation matrix construction

All statistical analyses were conducted using R software (version 4.3.3). Body mass index (BMI) was calculated from height and weight before statistical analysis. Stone recurrence was defined as two or more stone episodes during follow-up (Stone_Group = 1), whereas patients with only one documented stone episode were classified as non-recurrent (Stone_Group = 0). Only patients with complete baseline metabolic evaluation and recurrence information were included; therefore, no missing data imputation was performed.

To minimize the influence of different measurement scales, continuous variables, including age, HbA1c, TG, HDL-C, LDL-C, serum uric acid, PTH, urinary α1-microglobulin, BMI, 24-h urinary oxalate, citrate, calcium, and HOMA-IR, were standardized using z-score normalization before regression analysis.

Multivariable logistic regression was performed to evaluate the associations between baseline physicochemical variables and stone recurrence. Stone_Group served as the dependent variable, and all standardized variables were entered as independent predictors. Odds ratios (ORs), 95% confidence intervals (95% CIs), and corresponding *P*-values were reported. Statistical significance was defined as a two-sided *P* value < 0.05.

To explore the relationships among metabolic variables and to assist subsequent subgroup analyses and variable selection, Pearson correlation coefficients were calculated among all continuous variables. The resulting correlation matrix was visualized as a heatmap using the corrplot package in R.

#### Subgroup analysis based on correlation results

To further explore factors potentially associated with urinary citrate, Pearson correlation analysis was performed to identify variables showing linear correlations with 24-h urinary citrate. Variables identified as being correlated with urinary citrate were subsequently selected as stratification factors for subgroup analyses.

Subgroup analyses were performed to evaluate whether the association between urinary citrate and stone recurrence differed across strata defined by these variables. These analyses were intended to assess the potential heterogeneity of the citrate–recurrence association across clinically relevant metabolic subgroups and to inform subsequent subgroup-specific model development.

#### Hyperuricemia subgroup analysis

Patients were stratified according to serum uric acid status to further evaluate the association between urinary citrate and stone recurrence under different metabolic conditions. Hyperuricemia was defined as a serum uric acid concentration > 420 μmol/L in men and > 360 μmol/L in women.

Based on these thresholds, all participants were categorized into either the hyperuricemia group (elevated uric acid levels) or the non-hyperuricemia group (normal uric acid levels). Multivariate logistic regression analyses were then performed separately in each subgroup.

Furthermore, an interaction term between serum uric acid and 24-h urinary citrate (serum uric acid × urinary citrate) was incorporated into the multivariable logistic regression model to assess whether serum uric acid modified the association between urinary citrate and stone recurrence.

#### Prediction model development and internal validation

A preliminary recurrence prediction model was developed within the hyperuricemia subgroup. The initial multivariable logistic regression model included 13 candidate predictors: HOMA-IR, age, HbA1c, triglycerides, HDL-C, LDL-C, serum uric acid, PTH, urinary α1-microglobulin, BMI, 24-h urinary oxalate, 24-h urinary citrate, and 24-h urinary calcium. Bidirectional stepwise variable selection based on the Akaike Information Criterion was used to derive a more parsimonious final model. Internal validation was performed using 10-fold cross-validation with the caret package in R, and model discrimination was assessed using the mean area under the receiver operating characteristic curve. No external validation cohort was available. The final model included age, triglycerides, BMI, PTH, and 24-h urinary citrate.

## Results

### Multivariable logistic regression and subgroup analysis

**Fig. 1:**
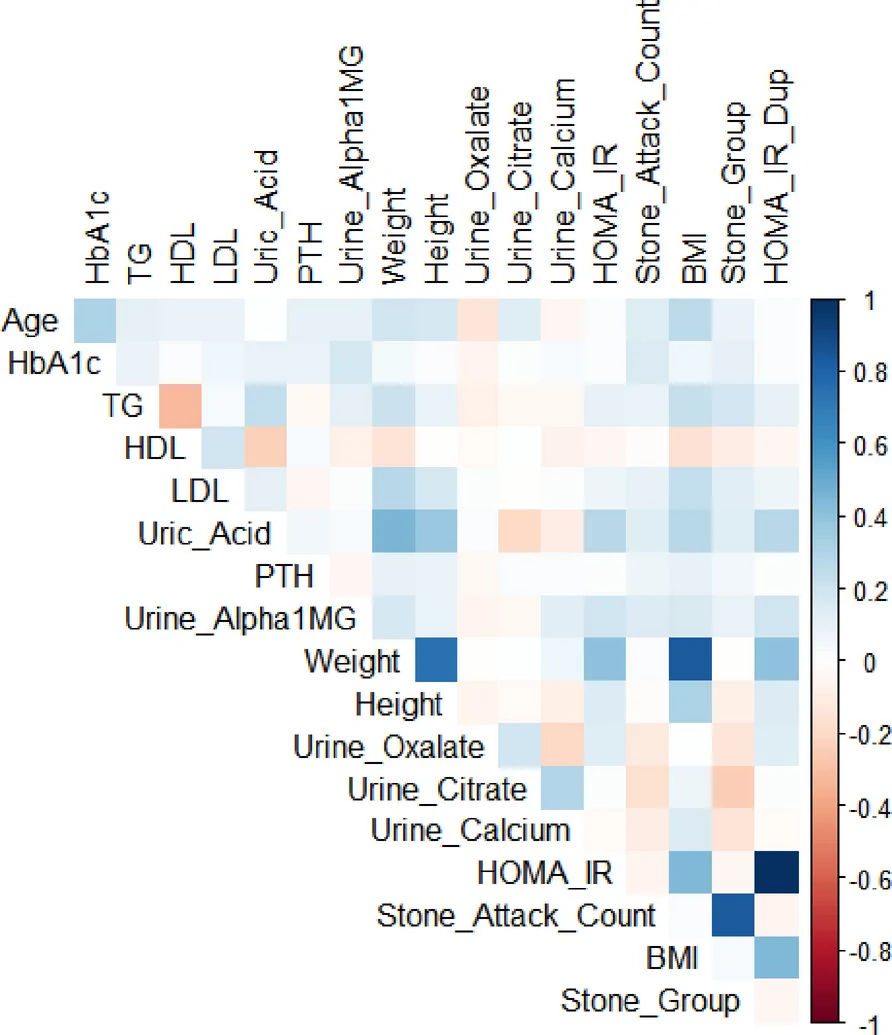
Pearson correlation matrix of demographic characteristics, serum biochemical parameters, urinary metabolic variables, and recurrence-related variables. The heatmap illustrates Pearson correlation coefficients among demographic characteristics, serum biochemical parameters, 24-h urinary metabolic variables, body mass index (BMI), HOMA-IR, stone attack count, and recurrence status (Stone_Group). Blue indicates positive correlations, red indicates negative correlations, and color intensity represents the strength of the correlation coefficient

Among all evaluated variables, only 24-h urinary citrate remained significantly associated with stone recurrence in the initial multivariable logistic regression analysis (Estimate = − 0.490, *P* = 0.037; Table 1). To further explore the relationships between urinary citrate and other metabolic variables, Pearson correlation analysis was performed using all continuous variables, and the resulting correlation matrix was visualized as a heatmap (Fig. 1).

**Table 1:**
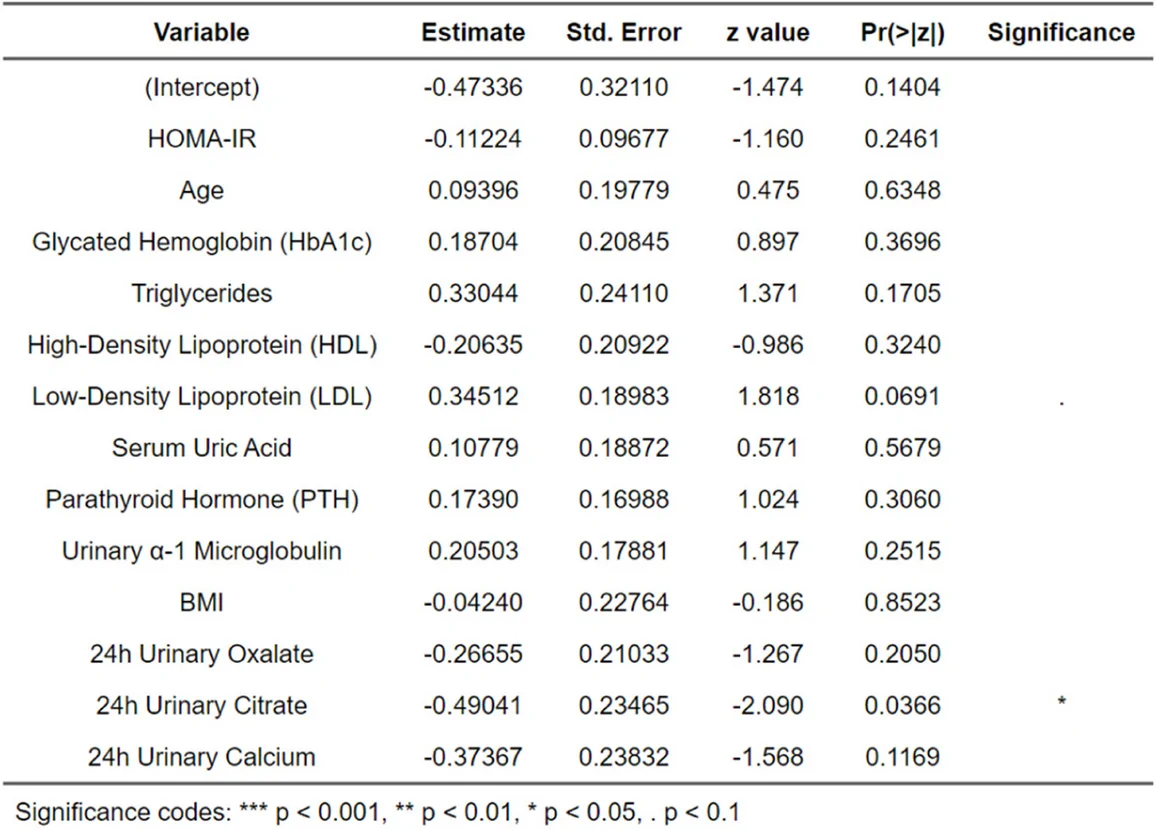
Multivariable logistic regression analysis of baseline metabolic parameters associated with urolithiasis recurrence in the overall study cohort

*Abbreviations*: *HOMA-IR* Homeostasis model assessment of insulin resistance, *HbA1c*, Glycated hemoglobin, *HDL* High-density lipoprotein, *LDL* Low-density lipoprotein, *PTH* Parathyroid hormone, *BMI* Body mass index

**Fig. 2:**
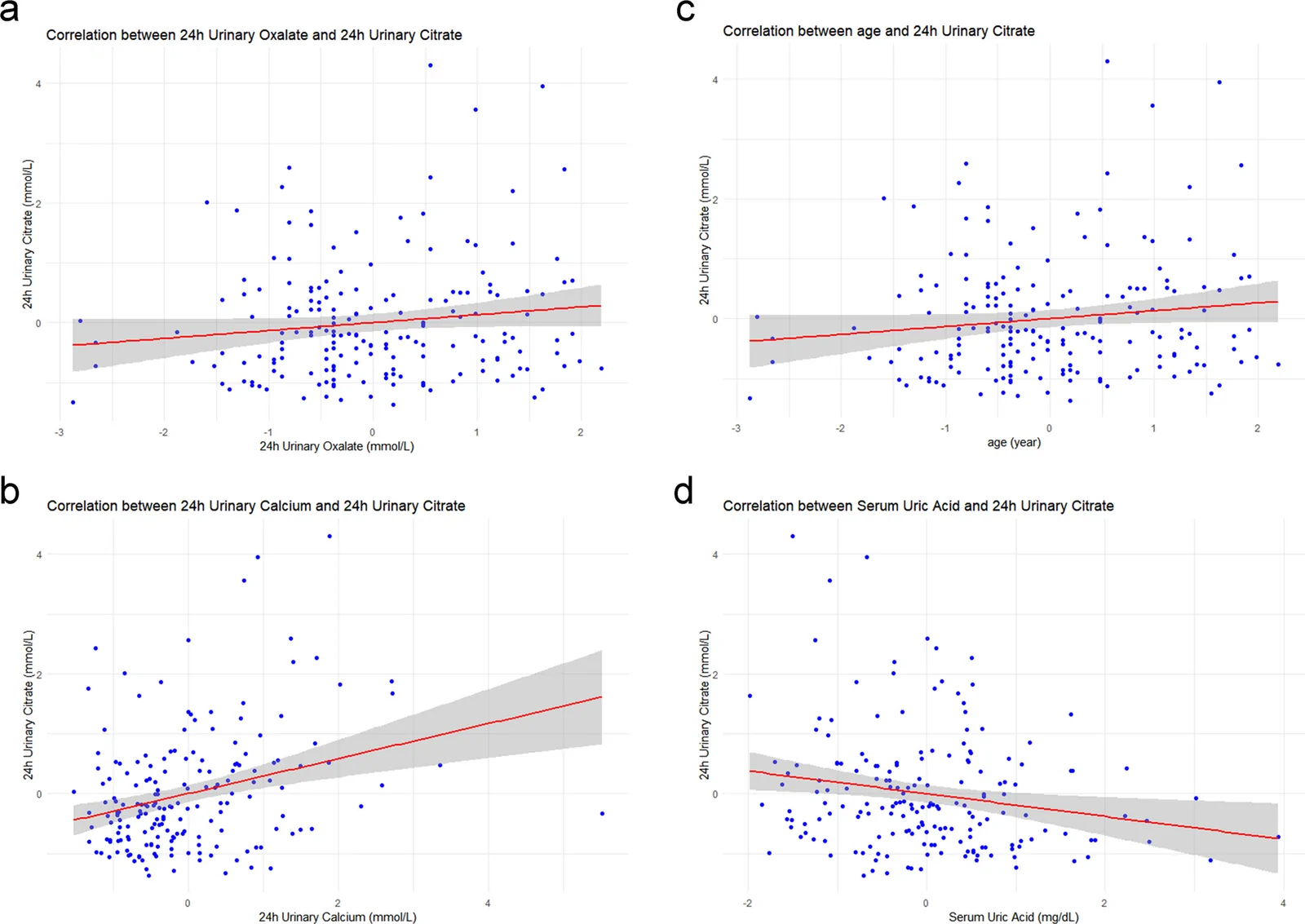
Correlations between 24-h urinary citrate and selected clinical variables. Scatter plots illustrating the relationships between 24-h urinary citrate and (**A**) 24-h urinary oxalate, (**B**) 24-h urinary calcium, (**C**) age, and (**D**) serum uric acid. The red line represents the fitted linear regression line, and the shaded area indicates the 95% confidence interval

Urinary citrate showed correlations with serum uric acid, 24-h urinary calcium, 24-h urinary oxalate, and age (Fig. 2A–D). Subgroup analyses were subsequently performed according to these correlated variables. A significant negative association between urinary citrate and stone recurrence was observed only in the hyperuricemia subgroup, whereas no statistically significant associations were identified in the remaining predefined subgroups.

In the hyperuricemia subgroup, urinary citrate remained significantly negatively associated with stone recurrence (Estimate = − 1.858, *P* = 0.034; Table 2A). In contrast, although the association remained negative in the non-hyperuricemia subgroup (Estimate = − 0.387, *P* = 0.135; Table 2B), it did not reach statistical significance.

**Table 2:**
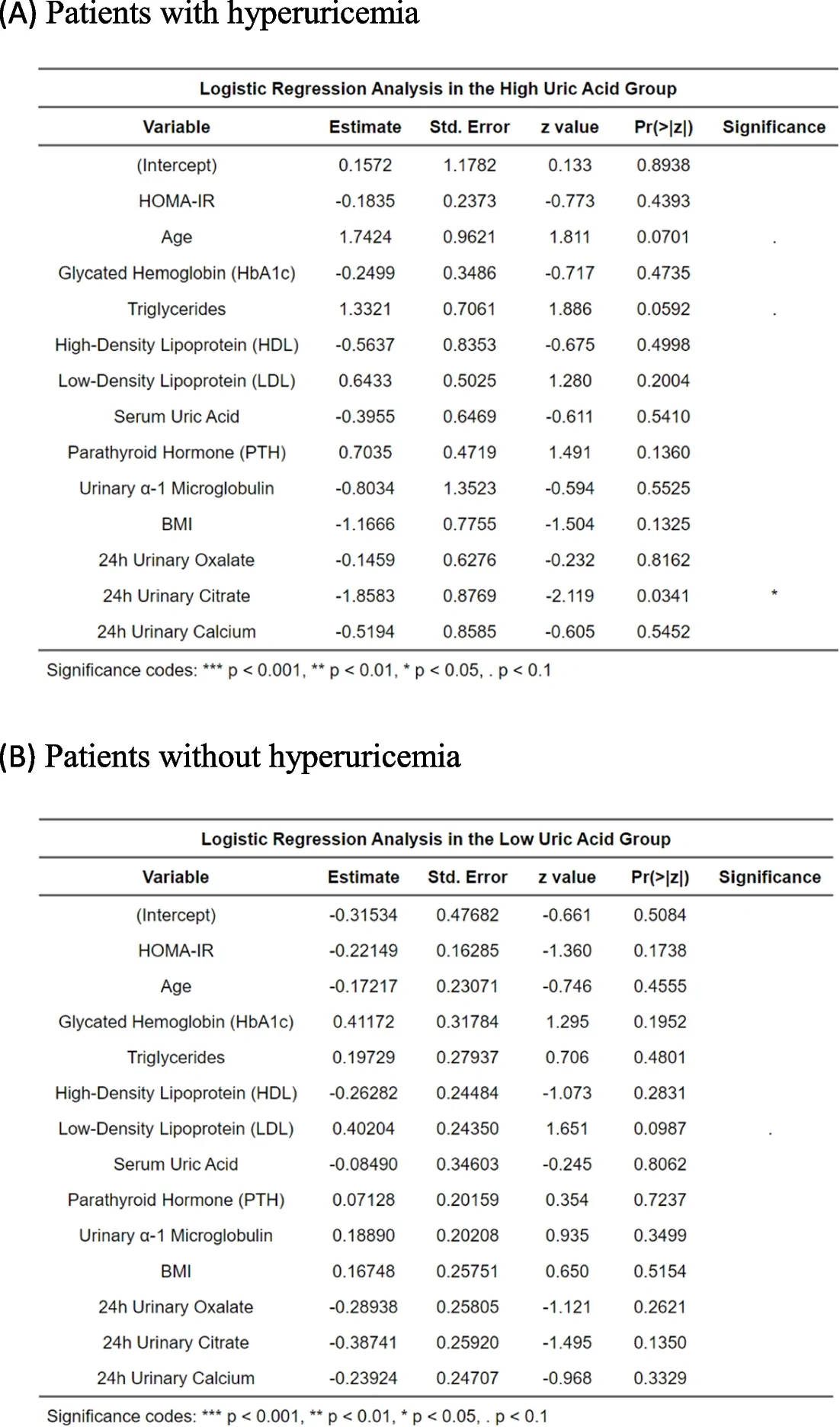
Multivariable logistic regression analyses stratified by hyperuricemia status

*Abbreviations*: *HOMA-IR* Homeostasis model assessment of insulin resistance, *HbA1c* Glycated hemoglobin, *HDL* High-density lipoprotein, *LDL* Low-density lipoprotein, *PTH* Parathyroid hormone, *BMI* Body mass index

An interaction term between serum uric acid and 24-h urinary citrate was incorporated into the multivariable logistic regression model. No statistically significant interaction between serum uric acid and urinary citrate was identified in either the hyperuricemia subgroup (Estimate = 1.01, *P* = 0.40) or the non-hyperuricemia subgroup (Estimate = − 0.27, *P* = 0.40).

### Model development and optimization

**Fig. 3:**
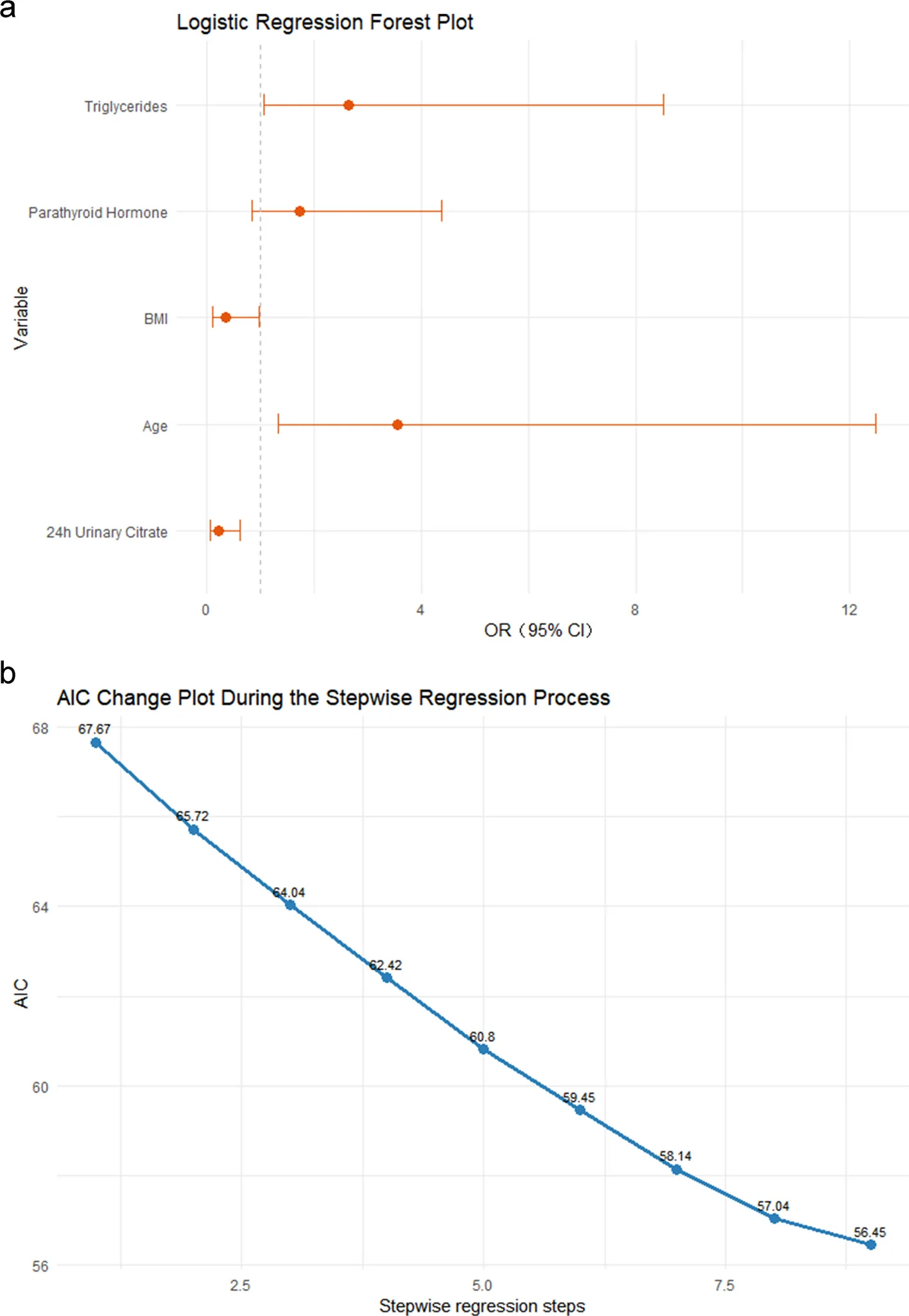
Development of the prediction model for urolithiasis recurrence in patients with hyperuricemia. **A** Forest plot showing the odds ratios (ORs) and 95% confidence intervals (CIs) of variables included in the final multivariable logistic regression model. The dashed vertical line indicates an OR of 1.0. **B** Changes in the Akaike Information Criterion (AIC) during bidirectional stepwise variable selection. Lower AIC values indicate improved model fit

Following the subgroup analyses, a subgroup-specific prediction model was developed within the hyperuricemia cohort. The initial multivariable logistic regression model included 13 candidate variables: HOMA-IR, age, HbA1c, triglycerides, HDL-C, LDL-C, serum uric acid, PTH, urinary α1-microglobulin, BMI, 24-h urinary oxalate, 24-h urinary citrate, and 24-h urinary calcium. Bidirectional stepwise regression based on the Akaike Information Criterion (AIC) was applied for variable selection. The final model retained five variables: age, triglycerides, PTH, BMI, and 24-h urinary citrate (AIC = 56.45; Fig. 3A–B).

**Fig. 4:**
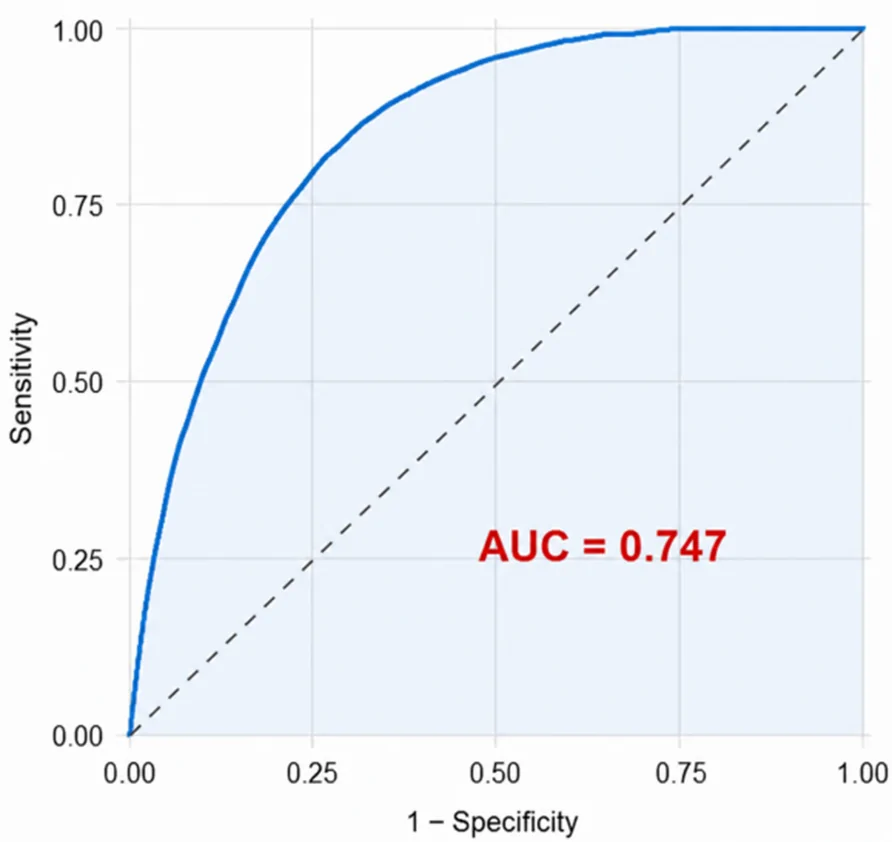
Receiver operating characteristic (ROC) curve of the final prediction model. Receiver operating characteristic (ROC) curve of the final prediction model developed for patients with hyperuricemia. Model performance was evaluated using tenfold cross-validation. The mean area under the curve (AUC) was 0.747, indicating moderate discriminative performance

In the initial 13-variable logistic regression model, the mean cross-validated accuracy was 56.7%, with a Cohen's kappa value of 0.126. After stepwise optimization, the final model achieved a mean cross-validated area under the receiver operating characteristic curve (AUC) of 0.747 (Fig. 4). The corresponding mean accuracy, sensitivity, and specificity were 71.4%, 68.3%, and 76.7%, respectively.

In the final multivariable logistic regression model, age (*P* = 0.023) and 24-h urinary citrate (*P* = 0.009) remained statistically significant predictors of stone recurrence, whereas triglycerides and BMI demonstrated borderline statistical significance (both *P* ≈ 0.078).

## Discussion

Urolithiasis is characterized by a high recurrence rate, and preventing recurrence remains one of the major challenges in stone management. Current preventive strategies, including increased fluid intake, dietary modification, and pharmacological therapy, are largely based on metabolic evaluation but are generally applied in a uniform manner rather than according to individual metabolic characteristics [9]. Although numerous urinary and serum metabolic parameters have been associated with stone formation, previous long-term studies have shown that most routine metabolic variables have limited value in predicting recurrence, whereas urinary citrate has consistently demonstrated a relatively stable association with recurrence risk. However, whether the predictive value of urinary citrate differs across distinct metabolic backgrounds has remained unclear.

Consistent with previous studies, urinary citrate was the only metabolic parameter independently associated with stone recurrence in the overall cohort. Subgroup analyses demonstrated that this association was significant only among patients with hyperuricemia. Although similar negative trends were observed in other metabolic subgroups, they did not reach statistical significance [6]. These findings suggest that the predictive value of urinary citrate may vary according to patients' metabolic background rather than being uniform across all stone formers.

Hyperuricemia is frequently accompanied by acidic urine, increased uric acid supersaturation, and metabolic abnormalities that favor urinary crystallization [10, 11]. Under these conditions, urinary citrate may exert a more prominent inhibitory effect through urinary alkalinization and calcium complex formation. Although the present study cannot establish causality, these mechanisms may partly explain why urinary citrate showed a stronger association with recurrence in hyperuricemic patients than in other metabolic subgroups.

Based on these findings, we developed a preliminary subgroup-specific prediction model for patients with hyperuricemia. The final model incorporated urinary citrate, age, BMI, triglycerides, and PTH and demonstrated moderate discriminative performance after internal cross-validation. Rather than proposing a universally applicable prediction tool, this exploratory model provides preliminary evidence that recurrence prediction may be improved by considering metabolic heterogeneity.

Age was a significant predictor (*P* = 0.023), potentially reflecting age-related decline in renal tubular function and decreased excretion of crystallization inhibitors such as citrate and magnesium [12]. Age also correlates with a higher burden of metabolic disturbances, such as insulin resistance and dyslipidemia, which are known contributors to stone formation [13–15].

Triglycerides, although marginally significant (*P* ≈ 0.078), may reflect underlying metabolic dysregulation. Hypertriglyceridemia, often a component of metabolic syndrome, is associated with increased stone risk via mechanisms including reduced urine pH and altered urinary calcium and oxalate excretion [16, 17]. Similarly, increased BMI has been linked to elevated urinary excretion of stone-promoting factors and reduced citrate and urine pH, creating a lithogenic milieu [18, 19].

PTH, although remaining within the normal reference range in most patients, was retained in the final model. This finding may indicate that subtle variations in calcium-phosphate metabolism are associated with recurrence risk, although the underlying mechanisms require further investigation [20].

Urinary citrate is already included in routine metabolic evaluation and potassium citrate is recommended for selected stone formers [21]. Our findings suggest that particular attention to hypocitraturia among patients with hyperuricemia may facilitate risk stratification. However, the present study should not be interpreted as evidence supporting prophylactic citrate therapy, and prospective interventional studies are required before therapeutic recommendations can be strengthened.

This study has several limitations. First, stone composition was unavailable because stone analysis was not routinely performed in all patients at the time of initial presentation. Consequently, we were unable to determine whether the association between urinary citrate and recurrence differed among different stone types. Second, this was a single-center retrospective study with a relatively small sample size, particularly after subgroup stratification. Therefore, the subgroup analyses and prediction model should be regarded as exploratory and require external validation in larger multicenter cohorts. Third, recurrence was primarily determined using standardized follow-up questionnaires rather than a predefined imaging surveillance protocol, which may have introduced recall and detection bias. Finally, only internal validation was performed because an independent validation cohort was unavailable.

In conclusion, urinary citrate remained the only metabolic parameter independently associated with stone recurrence in the overall cohort, with this association being particularly evident among patients with hyperuricemia. These findings support further investigation of metabolically stratified approaches to recurrence prediction and provide a basis for future prospective validation studies.

## Conclusion

This study demonstrated that urinary citrate was the only metabolic parameter independently associated with stone recurrence in the overall cohort, with this association being particularly evident among patients with hyperuricemia. A preliminary prediction model incorporating urinary citrate, age, BMI, triglycerides, and PTH showed moderate discriminative performance after internal validation. These findings suggest that metabolically stratified approaches may improve recurrence risk assessment and provide a basis for future prospective studies and external validation.

## Supplementary Information

Supplementary Material 1.

## Data Availability

The datasets generated and/or analyzed during the current study are available from the corresponding author on reasonable request.
